# Supplementary material for: Molecular Ancestry Across Allelic Variants of SLC22A1, SLC22A2, SLC22A3, ABCB1, CYP2C8, CYP2C9, and CYP2C19 in Mexican-Mestizo DMT2 Patients
Source: Biomedicines. 2025 May 9;13(5):1156. doi: 10.3390/biomedicines13051156 (PMC12109360; doi:10.3390/biomedicines13051156)
Supplement: Supplementary file 1 [file biomedicines-13-01156-s001.zip › Table S1.pdf]

Table S1. Analysis of allelic variants with their respective Taqman PCR-RT assays, enzymatic activity assignment, and activity score in DMT2 Mexican patients (n= 248).

Gen	rs ID	Nucleotide change	Activity/ Score	Activity	Taqman Assay ID
SLC22A1	rs12208357	c.181C>T			C__30634096_10
	rs2282143	c.1022C>T			C__15877554_40
	rs594709	c.839+597G>A			C___1898206_10
	rs622342	c.1386-2964C>A			C____928527_20
	rs628031	c.1222A>C			C___8709275_60
	rs683369	c.480G>T			C____928536_30
	rs72552763	c.1260_1262del			C__34211613_10
SLC22A2	rs316019	c.808T>G			C___3111809_20
SLC22A3	rs2076828	*698C>G			C___2763995_1_
	rs8187725	c.806C>A			C__30633894_10
ABCB1	rs1128503	c.1236C>T			C_7586662_10
	rs2032582	2677G>T/A			C_11711720C_30<br>C_11711720D_40
	rs1045642	c.3435T>A			C_7586657_20
CYP2C8					
**3*	rs11572080	c.2130G>A	*Decreased / 0.5*		C__25625794_10
**4*	rs1058930	c.11041C>G	*Decreased / 0.5*		C__25761568_20
CYP2C9					
**2*	rs1799853	c.430C>T	*Decreased / 0.5*		C_25625805_10
**3*	rs1057910	c.1075A>C	*None / 0*		C_27104892_10
**6*	rs9332131	c.818delA	*None / 0*		C__32287221_20
CYP2C19					
**2*	rs4244285	c.19154G>A	*None / 0*		C__25986767_70
**3*	rs4986893	c.17948 G>A	*None / 0*		C__27861809_10
**4*	rs28399504	c.1A>G	*None / 0*		C__30634136_10
**5*	rs56337013	c.1297C>T	*None / 0*		C__27861810_10
**17*	rs12248560	c.-806C>T	*Increased / 1.5*		C_ __469857_10
