# Supplementary material for: Molecular Ancestry Across Allelic Variants of SLC22A1, SLC22A2, SLC22A3, ABCB1, CYP2C8, CYP2C9, and CYP2C19 in Mexican-Mestizo DMT2 Patients
Source: Biomedicines. 2025 May 9;13(5):1156. doi: 10.3390/biomedicines13051156 (PMC12109360; doi:10.3390/biomedicines13051156)
Supplement: Supplementary file 1 [file biomedicines-13-01156-s001.zip › Table S2.pdf]

Table S2. Allelic and genotypic frequencies and activity score distribution by *CYP2C8*, *CYP2C9*, and *CP2C19* within a sample of Mexican DMT2 patients (n= 248).

	Genotypic frequency n= 248		Allele Frequency		Activity score	
Gen/SNV		n (%)		(%)		n (%)
CYP2C8 [a]	*1/*1	215 (87.75)	*1	93.67	1	1 (0.40)
	*1/*3	20 (8.16)	*3	4.28	1.5	29 (11.83)
	*1/*4	9 (3.67)	*4	2.04	2	215 (87.75)
	*3/*4	1 (0.40)				
rs11572080	GG	224 (90.32)	G	95.16		
	GA	22 (9.67)	A	4.84		
	AA	0 (0.00)	*p* ǂ	0.423		
rs1058930	CC	238 (93.01%)	C	97.98		
	CG	10 (4.03)	G	2.02		
	GG	0 (0.00)	*p* ǂ	0.745		
CYP2C9	*1/*1	217 (87.50)	*1	93.34	0.5	1 (0.40)
	*1/*2	16 (6.45)	*2	3.83	1	14 (5.64)
	*1/*3	13 (5.24)	*3	2.82	1.5	16 (6.45)
	*2/*2	1 (0.40)			2	217 (87.50)
	*2/*3	1 (0.40)				
rs1799853	CC	230 (92.74)	C	96.17		
	CT	17 (6.85)	T	3.83		
	TT	1 (0.40)	*p* ǂ	0.279		
rs1057910	AA	234 (94.35)	A	97.18		
	AC	14 (5.64)	C	2.82		
	CC	0 (0.00)	*p* ǂ	0.647		
rs9332131	AA	248 (100)	A	100		
	delA	0 (0.00)	delA	0		
	deldel	0 (0.00)	*p* ǂ	-		
CYP2C19	*1/*1	177 (71.37)	*1	84.27	0 PM	2 (0.80)
	*1/*2	36 (14.51)	*2	8.66	1	37 (14.91)
	*1/*4	1 (0.40)	*4	0.02	1.5	3 (1.20)
	*1/*17	27 (10.88)	*17	6.85	2	177 (71.37)
	*2/*2	2 (0.80)			>2 UM	29 (11.69)
	*17/*17	2 (0.80)				
	*2/*17	3 (1.20)				
rs4244285	GG	207 (83.46)	G	91.33		
	GA	39 (15.72)	A	8.67		
	AA	2 (0.80)	*p* ǂ	0.913		
rs4986893	GG	248 (100)	G	100		
	GA	0 (0.00)	A	0		
	AA	0 (0.00)	*p* ǂ	-		
rs28399504	AA	247 (99.59)	A	99.80		
	AG	1 (0.40)	G	0.20		
	GG	0 (0.00)				
rs56337013	CC	248 (100)	C	100		
	CT	0 (0.00)	T	0		
	TT	0 (0.00)	*p* ǂ	-		
rs12248560	CC	216 (87.09)	C	93.15		
	CT	30 (12.09)	T	6.85		
	TT	2 (0.80)	*p* ǂ	0.406		

[a]Undetermined (n=3), ǂp value for Pearson's Chi-square test determining Hardy-Weinberg equilibrium.
