# Supplementary material for: Molecular Ancestry Across Allelic Variants of SLC22A1, SLC22A2, SLC22A3, ABCB1, CYP2C8, CYP2C9, and CYP2C19 in Mexican-Mestizo DMT2 Patients
Source: Biomedicines. 2025 May 9;13(5):1156. doi: 10.3390/biomedicines13051156 (PMC12109360; doi:10.3390/biomedicines13051156)
Supplement: Supplementary file 1 [file biomedicines-13-01156-s001.zip › Table S3.pdf]

Table S3. SNV allelic and genotypic frequencies in *SLC22A1*, *SLC22A2*, *SLC22A3*, and *ABCB1* (n= 248).

Gene	SNV	Genotype	n (%)	Allele	Frequency (%)	*p*
		GAT/GAT	117 (47.17)	GAT	68.54	
	rs72552763	GAT/del	106 (42.74)	del	31.45	0.954
		del/del	25 (10.08)			
		A/A	106 (42.74)	A	64.11	
	rs622342	A/C	106 (42.74)	C	35.88	0.116
		C/C	36 (14.51)			
		CC	240 (96.77)	C	98.39	
	rs12208357	CT	8 (3.22)	T	1.61	0.796
		TT	0 (0.00)			
		CC	225 (90.72)	C	95.16	
SLC22A1	rs2282143	CT	22 (8.87)	T	4.84	0.563
		TT	1 (0.40)			
		AA	181 (72.98)	A	85.69	
	rs594709	AG	63 (25.40)	G	14.31	0.575
		GG	4 (1.61)			
		CC	223(89.91)	C	94.96	
	rs683369	CG	25 (10.08)	G	5.04	0.403
		GG	0 (0.00)			
		GG	179 (72.46)	G	85.22	
	rs628031[a]	GA	63 (25.50)	A	14.78	0.842
		AA	5 (2.02)			
		C/C	224 (90.32)	C	95.16	
SLC22A2	rs316019	C/A	24 (9.67)	A	4.83	0.198
		A/A	0 (0.00)			
		C/C	177 (71.37)	C	84.47	
	rs2076828	C/G	65 (26.20)	G	15.52	0.578
SLC22A3		G/G	6 (2.41)			
		CC	248 (100)	C	100	
	rs8187725	CT	0 (0.00)	T	0	-
		TT	0 (0.00)			
		C/C	59 (23.79)	C	48.58	
	rs1128503	C/T	123 (49.59)	T	51.41	0.486
		T/T	66 (26.61)			
		G/G	69 (28.16)	G	50.27	
		G/A	19 (7.75)	A	5.63	
ABCB1	rs2032582[b]	A/A	1 (0.40)	T	44.08	0.091
		G/T	105 (42.85)			
		T/T	39 (15.91)			
		T/A	12 (4.89)			
		C/C	70 (28.22)	C	56.25	0.028*
	rs1045642[d]	C/T	139 (56.04)	T	43.75	
		T/T	39 (15.72)			

[a]Undetermined (n=1), [b] missing (n= 3), ǂp value for Pearson's Chi-square test determining Hardy-Weinberg equilibrium. * Statistical significance ($p<0.05$).
