# Supplementary figures and images for: Molecular Ancestry Across Allelic Variants of SLC22A1, SLC22A2, SLC22A3, ABCB1, CYP2C8, CYP2C9, and CYP2C19 in Mexican-Mestizo DMT2 Patients
Source: Biomedicines. 2025 May 9;13(5):1156. doi: 10.3390/biomedicines13051156 (PMC12109360; doi:10.3390/biomedicines13051156)

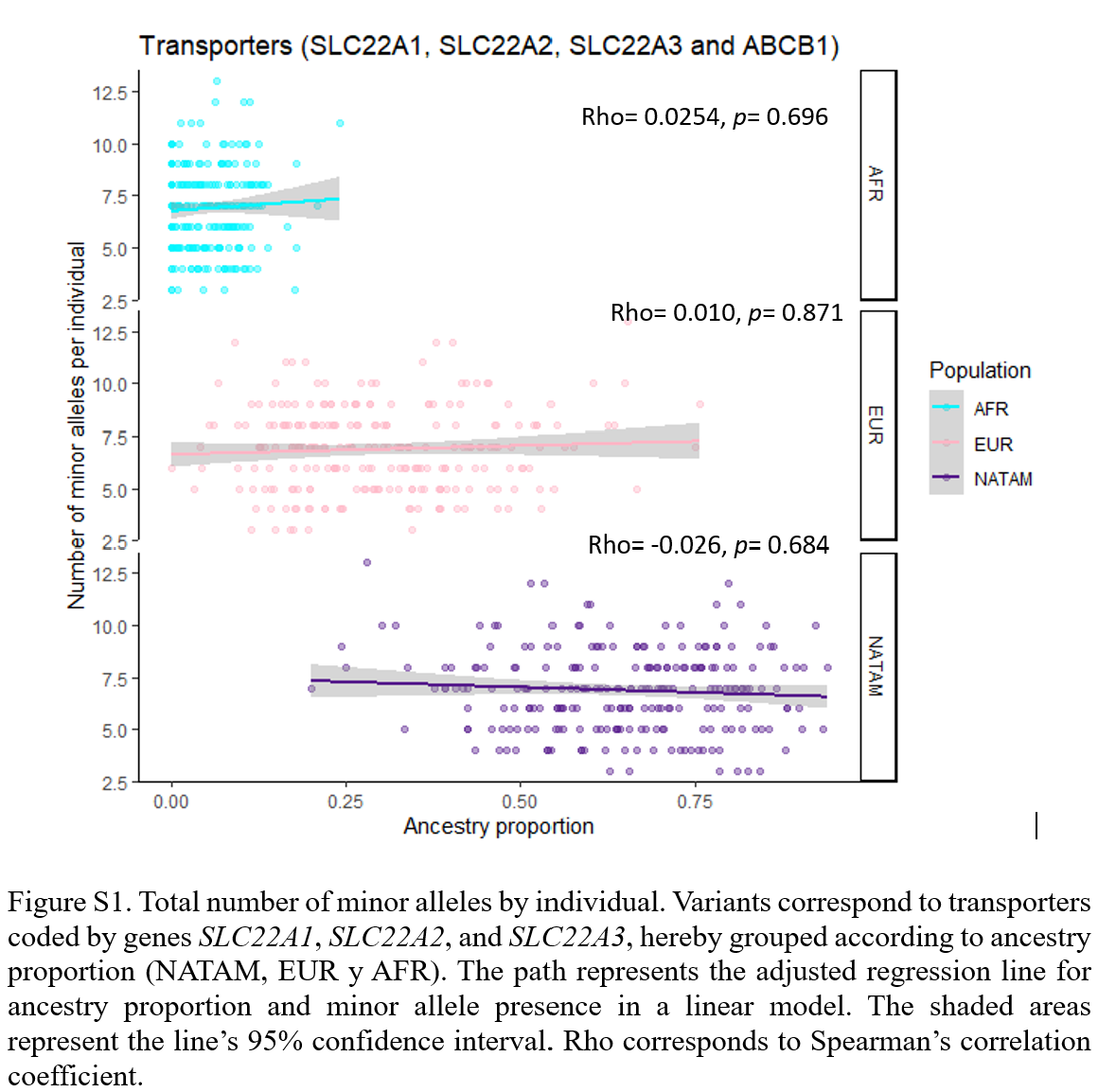

Supplement: Supplementary file 1 [file biomedicines-13-01156-s001.zip › Figure S1.png]

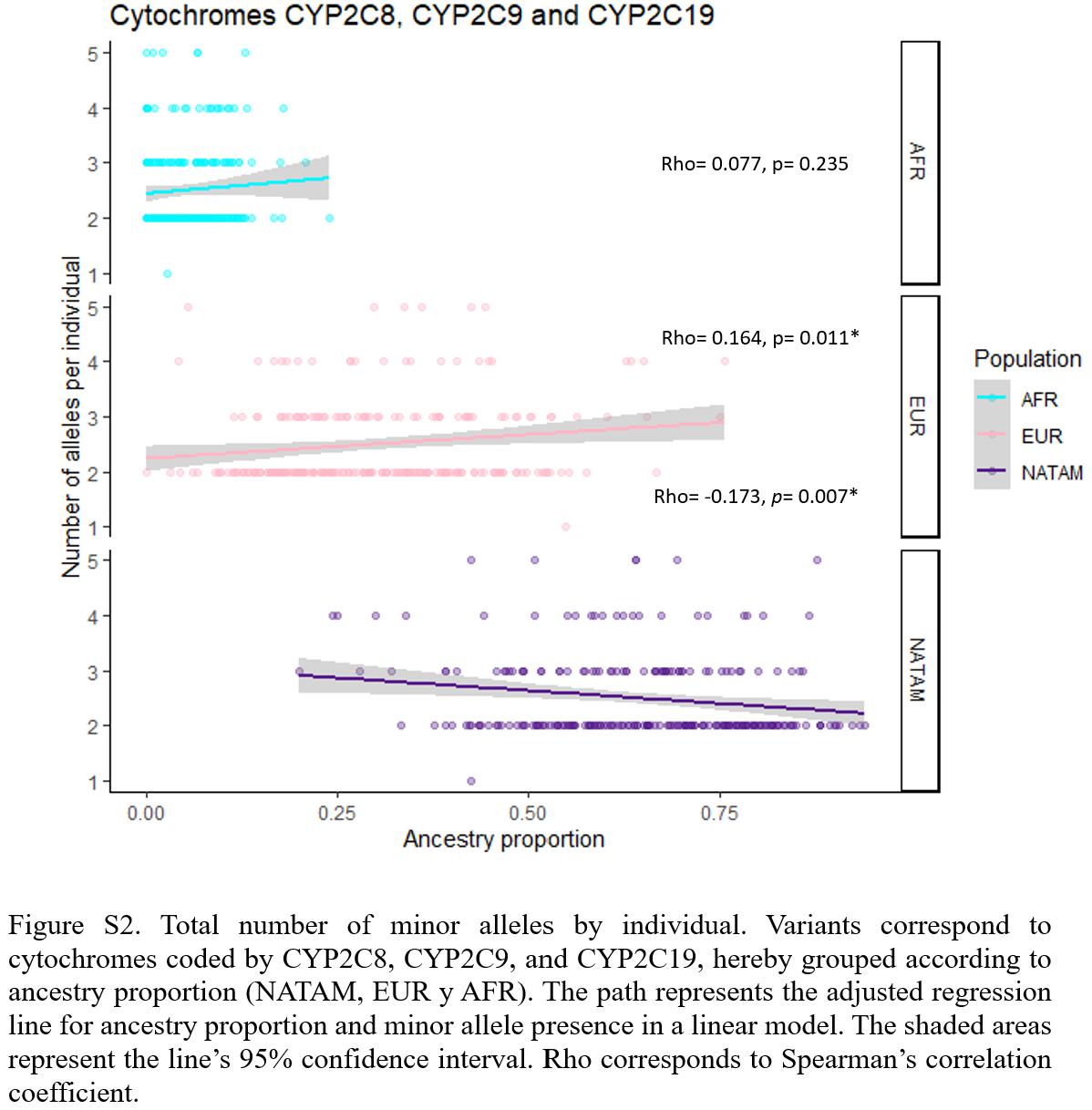

Supplement: Supplementary file 1 [file biomedicines-13-01156-s001.zip › Figure S2.png]
